# Productivity, impact, and collaboration differences between transdisciplinary and traditionally trained doctoral students: A comparison of publication patterns

**DOI:** 10.1371/journal.pone.0189391

**Published:** 2017-12-15

**Authors:** Anna-Sigrid Keck, Stephanie Sloane, Janet M. Liechty, Barbara H. Fiese, Sharon M. Donovan

**Affiliations:** 1 Division of Nutritional Sciences, University of Illinois at Urbana-Champaign, Urbana, Illinois, United States of America; 2 Illinois Transdisciplinary Obesity Prevention Program (I-TOPP), University of Illinois at Urbana-Champaign, Urbana, Illinois, United States of America; 3 Family Resiliency Center, University of Illinois at Urbana-Champaign, Urbana, Illinois, United States of America; 4 Department of Human Development and Family Studies, University of Illinois at Urbana-Champaign, Urbana, Illinois, United States of America; 5 School of Social Work, University of Illinois at Urbana-Champaign, Urbana, Illinois, United States of America; 6 College of Medicine, University of Illinois at Urbana, Urbana, Illinois, United States of America; OSWALDO CRUZ FOUNDATION, BRAZIL

## Abstract

Transdisciplinary (TD) approaches are increasingly used to address complex public health problems such as childhood obesity. Compared to traditional grant-funded scientific projects among established scientists, those designed around a TD, team-based approach yielded greater publication output after three to five years. However, little is known about how a TD focus throughout graduate school training may affect students’ publication-related productivity, impact, and collaboration. The objective of this study was to compare the publication patterns of students in traditional versus TD doctoral training programs. Productivity, impact, and collaboration of peer-reviewed publications were compared between traditional (n = 25) and TD (n = 11) students during the first five years of the TD program. Statistical differences were determined by t-test or chi square test at p < 0.05. The publication rate for TD students was 5.2 ± 10.1 (n = 56) compared to 3.6 ± 4.5 per traditional student (n = 82). Publication impact indicators were significantly higher for TD students vs. traditional students: 5.7 times more citations in Google Scholar, 6.1 times more citations in Scopus, 1.3 times higher journal impact factors, and a 1.4 times higher journal *h-index*. Collaboration indicators showed that publications by TD students had significantly more co-authors (1.3 times), and significantly more disciplines represented among co-authors (1.3 times), but not significantly more organizations represented per publication compared to traditional students. In conclusion, compared to doctoral students in traditional programs, TD students published works that were accepted into higher impact journals, were more frequently cited, and had more cross-disciplinary collaborations.

## Introduction

Transdisciplinary (TD) approaches are an increasingly common set of methods used to address complex global problems [1]. Approaches in TD, including team science, promote working both horizontally across traditional disciplines, professions, and stakeholders and vertically from cell to society to create new methods, processes, and practical solutions to grand challenges [2–4]. Thus, TD programs go beyond multi- and interdisciplinary approaches to foster synthesis across disciplines and focus on translating research findings into real world applications [2,5]. TD approaches have become more common in graduate training. However, formal evaluation of how TD training affects publication productivity, impact, and research collaboration is still needed [6,7].

Previous studies looking at publication productivity of established scientific teams at large research centers found that scientists who were part of TD/team science had an initial delay in scientific productivity [8–10]. They compared publication productivity of three different types of research teams that were awarded R01 grants; R01 is the most common NIH grant program, generally awarded for 3–5 years with no specific dollar limit. During the first three years, TD scientists produced fewer total publications than scientists with stacked or longitudinal NIH investigator initiated R01 grants and it took five years for TD scientists to produce a similar number of total publications as scientists with longitudinal R01 grants. However, after the initial lag period of 3–5 years (during which time TD teams were formed), the publication productivity of TD scientists outpaced the other two groups and this acceleration of productivity continued at year 10 [10]. Furthermore, TD scientists had significantly more co-authors per publication compared to scientists with long-term R01 grants (6.0 vs. 4.0, except for years 1 and 10), while the average impact factor of journals in which the team published their research across the full 10-years did not differ significantly among the three groups. To our knowledge, there are no data published that examine whether a TD focus during graduate school results in delayed student publication productivity, impact, or collaboration.

Therefore, this study was designed to test whether publication productivity, impact, and collaboration is different among doctoral students trained in TD science early in their career development compared to their peers in traditional doctoral programs. Primary research questions were: (1) Are there differences in publication productivity, impact, or collaboration between doctoral students in a TD training program and those in a traditional program within similar academic units? (2) Is the delay in publication productivity observed among established scientists who engage in TD research averted when TD training begins during doctoral training? (3) Does openness to collaboration as measured by the Interdisciplinary Perspectives Index and student demographics at the time of enrollment predict overall productivity, collaboration, or impact across groups?

## Materials and methods

### Research setting

This study was part of a larger evaluation of a federally-funded Illinois Transdisciplinary Obesity Prevention Program (I-TOPP), a TD doctoral training program. This study was approved by the University’s Institutional Review Board and informed consent was obtained prior to participation. The program started in 2011 with the aim of training future leaders in childhood obesity prevention through I-TOPP, a joint PhD/Masters of Public Health (MPH) degree program. The TD students spend their first two years completing the MPH course work and practicum experience, participating in research, and completing their qualifying exams. During the next three years, the TD students focus on doctoral courses, required courses in TD approaches to childhood obesity prevention, and their TD dissertation research under the guidance of mentors representing two or more disciplines. In addition, I-TOPP supports cross-disciplinary interactions with national and international leaders in childhood obesity prevention through a visiting faculty program, lecture series, and biennial symposium. The design and structure of this TD program allows students to develop depth in their specific discipline and TD breadth. The TD students enroll in both a traditional doctoral program for which they are required to comply with all department specific doctoral degree requirements, and they enroll in the I-TOPP program where they simultaneously explore methods and knowledge from other disciplines through additional I-TOPP courses, seminars, and MPH requirements. Thus, the structure of I-TOPP fosters the development of a professional disciplinary identity that is enhanced by multidisciplinary methods and theories, thereby laying a foundation for TD thinking and practice.

### Participants

Participants included all doctoral students enrolled in the TD pre-doctoral fellowship I-TOPP training program (n = 11) and traditional doctoral students (n = 25) also enrolled as full-time students during the same years (fall 2011, fall 2012 or fall 2013). Traditional students were drawn from the same academic units that participate in I-TOPP at a leading land-grant research university in the U.S. The TD students met the same departmental requirements as the traditional students, in addition to I-TOPP requirements.

The five participating departments represent disciplines relevant to obesity and human health: Food Science and Human Nutrition, Human Development and Family Studies, Kinesiology and Community Health, Division of Nutritional Sciences, and School of Social Work, all of which have been training doctoral students for decades. All departments require students to complete 96 hours of post-bachelor degree work with a mix of courses and research, a minimum GPA of 3.0 during graduate school, a preliminary exam, final defense and dissertation. Four of the five departments also requires all graduate students to pass a qualifying exam to ensure a basic knowledge in the disciplinary understanding. In addition, as part of the I-TOPP program, TD students were required to: (a) Complete an MPH degree including courses, capstone and practicum; (b) Work with a primary and secondary I-TOPP faculty advisor; (c) Undergo an annual review of progress and goals; (d) Complete two TD courses in childhood obesity prevention; and (e) Attend a TD seminar each semester taught by I-TOPP faculty from various disciplines and invited speakers. Table 1 shows the demographics and other characteristics of the students from both groups at time of enrollment as well as advisor characteristics.

**Table 1:** Sample characteristics of students in transdisciplinary (TD) and traditional doctoral programs at time of enrollment and advisor characteristics at program year 5.

	TD students (n = 11)	Traditional students (n = 25)	P-value	X^2^
	Mean +/- SD or n (%) [range]	Mean +/- SD or n (%) [range]		
Year of enrollment				
2011	3 (27)	7 (28)		
2012	3 (27)	5 (20)		
2013	5 (46)	13 (52)		
Age in years	24.5 ± 3.1 [20–31]	27.5 ± 4.3 [23–38]	0.045*	
Undergraduate major				
Animal science		3		
Biology	1	4		
Family/ Consumer		1		
Finance/ Business	1	1		
Fine arts		1		
International studies	1			
Kinesiology/ Exercise	2	3		
Nutrition/ Food/ Dietetics	2	3		
Psychology	3	4		
Political science		1		
Social work/ Human services	1	4		
Gender				0.097
Women	10 (91)	16 (64)		
Men	1 (9)	9 (35)		
Race				0.544
White	7 (64)	20 (80)		
Asian	2 (18)	2 (8)		
Black/ African American		1 (4)		
Multi-racial	2 (18)	2 (8)		
Ethnicity				0.257
Non-Hispanic	8 (73)	22 (88)		
Hispanic	3 (27)	3 (12)		
Doctoral department/ unit				
FSHN		2 (8)		
HDFS	4 (36)	3 (12)		
KCH	3 (27)	8 (32)		
DNS	3 (27)	7 (28)		
SSW	1 (9)	5 (20)		
Advisor characteristics				
	(n = 11^a^)	(n = 25^a^)		
Years in tenure track	19.0 ± 9.1 [4–29]	18.4 ± 9.3 [6–40]	0.864	
*Productivity* (# pubs)	77.0 ± 51.4 [16–57]	68.4 ± 81.0 [7–387]	0.747	
*Impact* (h-index)	24.4 ± 12.3 [5–45]	19.8 ± 15.4 [3–55]	0.392	
*Collaboration* (# co-authors across all pubs)	123.7 ± 42.7 [25–150^b^]	92.8 ± 53.0 [5–150^b^]	0.076	

Abbreviations: FSHN, Food Science and Human Nutrition; HDFS, Human Development and Family Studies; KCH, Kinesiology and Community Health; DNS, Division of Nutritional Sciences; SSW, School of Social Work

^a^Number represents all primary advisor-student pairs to reflect overall faculty influence per student group. Two advisors in each of the groups (TD and traditional) had more than one student. In addition, one faculty member advised both a TD student and a traditional student.

^b^Maximum number of co-authors reported in Scopus analytics is 150.

*p ˂ 0.05

### Procedures/ Measures

#### Surveys

Interdisciplinary attitudes were measured by the Interdisciplinary Perspectives Index (IPI) survey, a 6-item scale with a 5-point Likert response from strongly agree to strongly disagree. This is a tool designed to assess openness to interdisciplinary perspectives [11]. In this study, all but one item were reverse coded so that higher values indicate more interdisciplinarity. Collaborative behaviors were measured by the Behavior Change Collaborative Activities Index (BCCAI), a 7-item scale with a 7-point Likert response from never to very often [11]. The IPI and BCCAI were completed by the students in both groups at the start of their doctoral program.

#### Productivity

Publication productivity was assessed via an objective search of peer-reviewed scientific articles (not including meeting abstracts) for each student from the time they started their doctoral program through August 2016. The following bibliometric sources were used to identify publications: PubMed, Scopus, Google Scholar, and University library and departmental websites. All published or in press peer-reviewed articles identified from one of these sources were included. Publication productivity indicators for each group included the average number of publications per student, percent of students with a first author publication, and percent of students with at least one publication. We also examined the number of different (unique) journals in which students published their work to assess the degree to which works by each group of students reached scientific audiences in a broad versus limited range of journals.

#### Impact

To assess impact of publications by each group, we used the following indicators: (a) the number of citations per publication in Scopus and Google Scholar; (b) journal impact factors from 2016 Journal Citation Reports^®^; and (c) the *h-index* (described below) of journals with student publications. All impact indicators were assessed as of August 2016, five years into the TD program. Not all publications found in Google Scholar were found in Scopus (51 of 56 for TD students and 74 of 82 for traditional students) due to the journal inclusion criteria in the Scopus database. The *h-index* for each journal was identified through SCImago Journal & Country Rank [12], a publicly available portal based on information contained in the Scopus database. The *h-index* expresses the number of articles (*h*) in a given journal that have received at least *h* citations, thus quantifying both scientific productivity and scientific impact at the journal level.

#### Collaboration

The average number of co-authors per publication, disciplines per publication, and organizations represented on each publication were compared between groups to assess collaboration. The disciplines per publication indicator is defined in this article as unique departments or units listed for the authors on each publication.

### Statistical analyses

Descriptive data, including demographics, were collected on all students in the study. Mean and standard deviations were calculated on each metric for each group and compared via 2-tailed independent samples t-tests for continuous measures and chi-square statistics for categorical measures using IBM SPSS 24.0. A p-value less than 0.05 was considered statistically different.

## Results

At baseline, students in TD and traditional groups differed only in age (Table 1) and on two of the seven items regarding collaboration on the BCCAI (Table 2). They did not differ by race, ethnicity, gender (Table 1) or interdisciplinarity on the IPI index (Table 3). At five years into the TD program, groups were assessed on publication productivity, impact, and collaboration based on previously described characteristics of their peer-reviewed publications (Table 4).

**Table 2:** Behavior Change Collaborative Activities Index (BCCAI) scores of students in transdisciplinary (TD) and traditional doctoral programs at time of enrollment.

Items	TD students (n = 11)	Traditional students (n = 18)	P-value
	Mean +/- SD	Mean +/- SD	
Read journals outside your field or major?	4.4 ± 1.4	5.1 ± 1.5	0.237
Attend conferences outside your field or major?	2.5 ± 1.8	3.2 ± 1.6	0.294
Participate in groups with researchers in other fields with the intent to integrate ideas?	3.2 ± 1.5	4.2 ± 1.5	0.101
Obtain new insights into your own area of research through discussion with other researchers (e.g., developed a new concept or hypothesis that bridges or integrates different disciplinary or theoretical approaches to your research)?	3.5 ± 1.6	4.7 ± 1.4	0.049*
Attempt to establish links with other interdisciplinary researchers that may lead to future collaborative studies?	4.5 ± 2.1	4.8 ± 1.4	0.618
Actually design a new collaborative study as a result of working on an ongoing interdisciplinary project?	1.8 ± 1.2	3.6 ± 1.8	0.009*
Take class outside your field or major?	5.2 ± 1.5	5.1 ± 1.6	0.835
*Mean BCCAI score*	*3*.*6 ± 1*.*4*	*4*.*4 ± 0*.*5*	0.162

Note. Questions 1–7 are on a scale from 1 (never) to 7 (very often).

*p ˂ 0.05

**Table 3:** Interdisciplinary Perspectives Index (IPI) scores of students in transdisciplinary (TD) and traditional doctoral programs at time of enrollment.

Items	TD students (n = 11)	Traditional students (n = 18)	P-value
	Mean +/- SD	Mean +/- SD	
In my own research, I typically use multiple research methods drawn from more than one discipline rather than rely exclusively on a single disciplinary approach.	3.4 ± 0.9	4.0 ± 0.8	0.071
I prefer to conduct research independently rather than as a part of a group.	3.7 ± 0.6	3.3 ± 1.0	0.266
I would describe myself as someone who strongly values interdisciplinary collaboration.	4.5 ± 0.5	4.2 ± 0.8	0.368
Generally speaking, I believe that the benefits of interdisciplinary research outweigh the inconvenience of such work.	4.2 ± 0.8	4.1 ± 0.6	0.633
I am optimistic that interdisciplinary collaboration among faculty will lead to valuable scientific outcomes that would not have occurred without that collaboration.	4.8 ± 0.4	4.5 ± 0.6	0.142
Overall, I believe that a high level of good will exists among the research associates at University of Illinois affiliated with my research.	4.7 ± 0.5	4.2 ± 0.7	0.051
*Mean IPI score*	*4*.*2 ± 0*.*4*	*4*.*0 ± 0*.*5*	*0*.*312*

Note. Questions are on a 1–5 scale; all items, except item #2, were recoded such that higher values reflect greater agreement with interdisciplinary perspectives.

**Table 4:** Group-level differences in publication patterns comparing students in transdisciplinary (TD) and traditional doctoral programs at year five of the TD program^a^.

Group-level differences	TD student publications (n = 56)	Traditional student publications (n = 82)	P-value	X^2^
	Mean +/- SD or n (%)	Mean +/- SD or n (%)		
*Productivity indicators*				
Total publications	56	82		
Publications per student	5.2 ± 10.1 [range 0–35]	3.6 ± 4.5 [range 0–17]	0.504	
Student with 1st author publication(s)	6/11^b^ (55)^c^	13/25^b^ (52)		0.888
Students with ≥ 1 publication	9/11^b^ (82)	17/25^b^ (68)		0.394
Publications in unique journals	41 (73)	73 (89)		0.016*
*Impact indicators*				
Google Scholar citations per publication	21.5 ± 37.5 [range 0–192] n = 56	3.8 ± 5.5 [range 0–33] n = 82	0.001**	
Scopus Scholar citations per publication	14.0 ± 22.1 (range 0–112) n = 51	2.3 ± 3.7 (range 0–26) n = 74	0.0001**	
Journal impact factor	3.3 ± 1.3 n = 53	2.6 ± 1.4 n = 72	0.006**	
Journal *h-index*^d^	110.4 ± 66.8 n = 53	81.5 ± 51.9 n = 75	0.010*	
*Collaboration indicators*				
Co-authors per publication	6.9 ± 2.9	5.4 ± 2.6	0.002**	
Disciplines per publication	3.4 ± 1.6	2.6 ± 1.5	0.003**	
Organizations per publication	2.3 ± 1.6	1.9 ± 1.0	0.105	

^a^At five years into the TD program all students in the sample were in year 3, 4 or 5 of their doctoral program.

^b^The number of students in each group was the denominator rather than number of publications.

^c^Percentages were based on a total of 56 publications for the 11 I-TOPP scholars and 82 publications for the 25 traditional PhD students.

^d^*h-index* at the journal level was extracted from the SCImago Journal & Country Rank, based on the Scopus^®^ database. The h-index expresses the journal’s number of articles (*h*) that have received at least *h* citations.

*p ˂ 0.05.

**p ˂ 0.001

### Productivity

**Fig 1:**
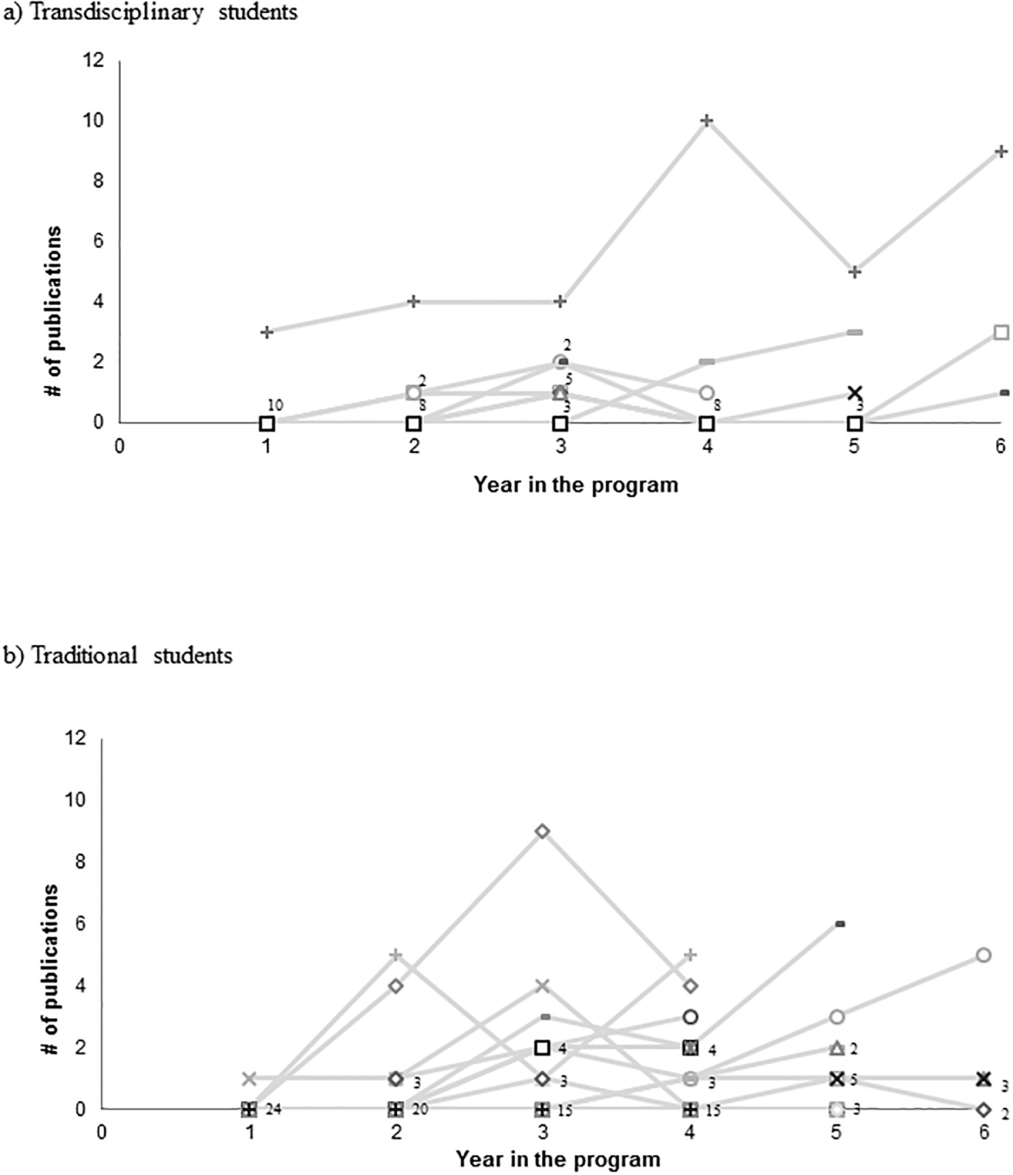
Publications per student per year in transdisciplinary (a; n = 11) compared to traditional (b; n = 25) doctoral training. Each line represents a student’s number of publications per year in the program (if other than 0 each year). Number labels are shown when multiple students have the same number of publications in a given year.

The group of TD students produced 5.2 ± 10.1 publications per student (56 total in 41 unique journals) compared to 3.6 ± 4.5 publications per student (82 total in 73 unique journals) produced by the traditional students, but this difference was not significant (p = 0.504). High performers from both groups were within two standard deviations and thus were retained (Fig 1). Both groups had a similar percentage of students who were first authors (52% and 55%, p = 0.888) and a similar percentage of students that had at least one publication (68% and 82%, p = 0.394).

Both groups published in a wide range of journals. However, compared to TD students, traditional students published a higher percentage of papers in unique journals (89% vs 73%; X^2^ = 0.016). Specifically, the 25 traditional students published a total of 82 papers in 73 journals (89% in unique journals) while the 11 TD students published a total of 56 papers in 41 journals (73% unique; Table 4).

### Impact

All four indicators of publication impact were significantly greater for the TD group than the traditional group (Table 4). First, the number of citations per publication in Google Scholar was 5.7 times higher (p = 0.001) for TD than traditional students. Second, the number of citations per publication using Scopus, which tracks a more select subset of publications (51 for TD and 74 for traditional) that are indexed in the major scientific databases, was 6.1 times higher (p < 0.001) for TD than traditional students. Finally, journal impact factor, obtained from 2016 Journal Citation Reports^®^ and journal *h-index*, were significantly higher (1.3-times and 1.4- times, respectively) for the publications by the TD group compared to the traditional group (p = 0.006 and p = 0.01, respectively).

### Collaboration

The TD student group had 1.3-times more co-authors per publication compared to the traditional group (p = 0.002). The number of disciplines represented in each publication was also significantly higher (1.3-times) for the TD group compared to the traditional group (p = 0.003). The number of organizations represented in each publication was not significantly different between the groups but did trend in a similar direction as the other two measures (p = 0.105).

## Discussion

To our knowledge this is the first study to objectively assess differences in publication productivity, impact, and collaboration between doctoral students pursuing a TD training program compared to doctoral students enrolled in traditional doctoral programs. It is important to note that the two groups differed only in size and age. There were 11 students participating in the I-TOPP TD program, and 25 traditional doctoral students enrolled in the same academic departments as the TD students. The traditional students were on average three years older than the TD students (Table 1). The groups differed on two of the seven collaboration items of the BCCAI, with the traditional group reporting greater collaborative behavior at baseline (Table 2). The groups did not differ on interdisciplinarity measured on the IPI at baseline (Table 3). This is an important finding since self-selection into each type of training is always a potential confound. Longitudinal findings on differences in the three attributes of scholarship are discussed below.

### Productivity

In response to research questions one and two, no significant differences in publication productivity by training type were found, suggesting there is no delay in publication productivity among TD trained students compared to traditional approaches, which had been observed among established scientists engaged in team science projects [10]. The publication productivity rates for both groups of students in this study (Table 4) were comparable to publication rates among doctoral students at other research universities in the U.S. [13,14]. Both TD and traditional groups in this study published in a wide range of journals rather than in a small select group of journals. Although one current trend in higher education is to question productivity as a primary or sole indicator of scholarly quality or success, and the value of “slow science” is being actively discussed [15,16], publication productivity is still one of the most frequently used objective measures for hiring, promotion, and tenure in academia. Moreover, other studies have reported a strong correlation between productivity and the number of citations among established researchers, though not necessarily among junior researchers [17,18]. In this study, no correlation between publication productivity and impact was found among TD or traditional students during their training period. Future studies are needed to assess if associations between publication productivity and impact differ long-term by type of training.

### Impact

In response to research question one, the TD trained students produced publications with significantly higher impact than the traditionally trained students on all four indicators: Scopus citations, Google Scholar citations, journal impact factors, and the *h-index* of unique journals in each group (Table 4). This finding was surprising because the two groups did not differ on productivity (number of publications), yet publication productivity and impact have been reported by others to be strongly correlated [17,18]. A possible explanation for this unexpected finding—that TD students had higher publication impact but did not differ on number of publications—may point to differences in training. The TD students’ public health and TD training was designed to foster a focus on research that addresses grand challenges, incorporates interdisciplinary perspectives, and offers practical recommendations and solutions based on their research findings; thus resulting in more frequently and timely cited publications in higher impact journals.

### Collaboration

In response to the first research question, students in the TD program had a greater number of co-authors and disciplines represented on their publications than the traditionally-trained doctoral students. The TD group also had more organizations per publication but not significantly more (Table 4). These findings are interesting because there were either no differences between groups on collaboration at baseline, or students in the traditional group actually perceived themselves as more collaborative than did the TD group at baseline (Table 2). The apparent heightened collaboration among TD students over time likely reflects the fact that the TD students were enrolled in a program that values and requires TD/team science approaches when designing and executing research projects. In addition, TD students also have co-advisors from two different disciplines, and are encouraged to reach out to researchers and students outside of their research group to conduct research projects. They also have frequent exposure to invited speakers and lectures through the TD program. This finding—greater collaborative behaviors among TD students over time—is consistent with a study that observed greater collaboration on publications for scientists on team science grants versus traditional R01 grants [10]. However, the validity of collaboration as an indicator of academic success or publication impact remains an open question for future research.

### Predictors of productivity, collaboration, and impact

In response to the third research question, regarding factors that might predict overall publication patterns during graduate school, none of the student demographics (including year of enrollment) or the measure of interdisciplinarity (IPI index) at baseline predicted productivity, impact, or the collaborative nature of publication patterns in either group or between groups based on regression analyses (not shown). This is consistent with the literature, which has found few, if any, predictors of productivity during graduate school [14]. It is also consistent with the philosophy that scholars are “made not born” [19] and highlights the importance of multi-dimensional characteristics of doctoral education (e.g., individual effort; interpersonal factors such as mentoring, supervision, and peer support; and institutional factors such as climate and infrastructure) [19]. In addition, student advisor characteristics were not significantly different between the two groups (Table 1), suggesting that advisor publication productivity, collaboration, and impact are not the main influence on student productivity, collaboration, and impact with regard to publication patterns. The greater impact and collaboration among TD students may be due to the nature of the I-TOPP program, described here and elsewhere [20], which fostered high support and high expectations, and provided highly structured programmatic support.

### Limitations

The main limitation of this study is the small sample size of the TD student group, a function of highly structured federally funded training programs; however, 100% of students in the TD program agreed to participate in this study. This longitudinal study is also limited to the 5-year period in which the TD program has been in place. The long-term impact of early TD compared to traditional doctoral training will require additional follow-up; we plan to continue data collection over time to address this limitation.

## Conclusions

This study examined differences between doctoral students in TD versus traditional training programs based on multiple indicators of publication productivity, impact, and collaboration. Compared to traditional education, a TD focus during doctoral training led to equal publication productivity by both groups and to significantly greater publication impact and collaboration across disciplines. Early TD training appears to stimulate the practice of high-impact team science. Further research is needed to determine if the publication-related benefits of early TD training will translate into long-term scientific productivity, collaboration, and impact.

## Supporting information

S1 File Donovan, Fiese, Liechty & Keck (2016), Journal of Nutrition Education and Behavior, 48, S118. http://dx.doi.org/10.1016/j.jneb.2016.04.343.
(PDF)

## References

[1] World Health Organization. World health statistics 2016: monitoring health for the SDGs, sustainable development goals. 2016 http://www.who.int/gho/publications/world_health_statistics/2016/en/. Cited 13 June 2017.

[2] Stokols D, Hall KL, Vogel AL. Transdisciplinary public health: definitions, core characteristics, and strategies for success In: Transdisciplinary public health: research, methods, and practice. San Francisco: Jossey-Bass; 2013: 3–30.

[3] Abrams DB. Applying transdisciplinary research strategies to understanding and eliminating health disparities. Health Educ Behav. 2006;33(4): 515–531. http://journals.sagepub.com/doi/abs/10.1177/1090198106287732. 16769758 10.1177/1090198106287732

[4] Townsend T, Pisapia J, Razzaq J. Fostering interdisciplinary research in universities: a case study of leadership, alignment and support. Studies in Higher Education. 2015;40(4): 658–675. 10.1080/03075079.2013.842218.

[5] Wall S, Shankar I. Adventures in transdisciplinary learning. Studies in Higher Education. 2015:40(4): 658–675. 10.1080/03075079.2013.842218.

[6] Mitrany M, Stokols D. Gauging the transdisciplinary qualities and outcomes of doctoral training programs. Journal of Planning Education and Research. 2005:24(4): 437–449. 10.1177/0739456X04270368.

[7] Nash JM. Transdisciplinary training: key components and prerequisites for success. Am J Prev Med. 2008:35(2): S133–S140. 10.1016/j.amepre.2008.05.004. 18619393

[8] Cooper LA, Boulware LE, Miller ER III, Golden SH, Carson KA, Gary Noronha G, et al Creating a transdisciplinary research center to reduce cardiovascular health disparities in Baltimore, Maryland: lessons learned. Am J Public Health. 2013:103(11): e26–e38. 10.2105/AJPH.2013.301297. 24028238 PMC3828697

[9] Hall KL, Stokols D, Moser RP, Taylor BK, Thornquist MD, Nebeling LC, et al The collaboration readiness of transdisciplinary research teams and centers findings from the National Cancer Institute’s TREC Year-One evaluation study. Am J Prev Med. 2008:35(2 Suppl): S161–172. 10.1016/j.amepre.2008.03.035. 18619396 PMC3292855

[10] Hall KL, Stokols D, Stipelman BA, Vogel AL, Feng A, Masimore B, et al Assessing the value of team science: a study comparing center- and investigator-initiated grants. Am J Prev Med. 2012;42(2): 157–163. 10.1016/j.amepre.2011.10.011. 22261212 PMC3586819

[11] Misra S, Harvey RH, Stokols D, Pine KH, Fuqua J, Shokair SM, et al Evaluating an interdisciplinary undergraduate training program in health promotion research. Am J Prev Med. 2009;36(4): 358–365. 10.1016/j.amepre.2008.11.014. 19201144

[12] SCImago. SJR—SCImago Journal & Country Rank. Published 2007. Cited 13 June 2017. http://www.scimagojr.com.

[13] National Research Council. A Data-Based Assessment of Research-Doctorate Programs in the United States. Washington, DC: The National Academies Press 2011 https://grants.nih.gov/training/research_doctorates.pdf. Cited 13 June 2017. 22379653

[14] Hall JD, O’Connell AB, Cook JG. Predictors of student productivity in biomedical graduate school applications. PloS One. 2017:12(1): e0169121 10.1371/journal.pone.0169121. 28076439 PMC5226343

[15] Hicks D, Wouters P, Waltman L, De Rijcke S, Rafols I. The Leiden Manifesto for research metrics. Nature. 2015;520(7548): 429–431. 10.1038/520429a. 25903611

[16] Wilsdon J, Allen L, Belfiore E, Cambell P, Curry S, Hill S, et al. The metric tide: report of the independent review of the role of metrics in research assessment and management. July 2015. http://blogs.lse.ac.uk/impactofsocialsciences/files/2015/07/2015_metrictide.pdf. 10.13140/RG.2.1.4929.1363. Cited 13 June 2017.

[17] Larivière V, Costas R. How many is too many? on the relationship between research productivity and impact. PloS one. 2016;11(9): e0162709 10.1371/journal.pone.0162709. 27682366 PMC5040433

[18] Sandström U, van den Besselaar P. Quantity and/or quality? the importance of publishing many papers. PloS one. 2016;11(11): e0166149 10.1371/journal.pone.0166149. 27870854 PMC5117611

[19] Liechty JM, Liao M, Schull CP. Facilitating dissertation completion and success among doctoral students in social work. J Soc Work Educ. 2009;45(3): 481–497. 10.5175/JSWE.2009.200800091.

[20] Keck A-S, Sloane S, Liechty JM, Paceley MS, Donovan SM, Bost KK, McBride BA, Fiese BH. Longitudinal Perspectives of Faculty and Students on Benefits and Barriers to Transdisciplinary Graduate Education: Program Assessment and Institutional Recommendations. Palgrave Commun. 2017;3(article 40). 10.1057/s41599-017-0027-y.

