# Supplementary material for: Productivity, impact, and collaboration differences between transdisciplinary and traditionally trained doctoral students: A comparison of publication patterns
Source: PLoS One. 2017 Dec 15;12(12):e0189391. doi: 10.1371/journal.pone.0189391 (PMC5731691; doi:10.1371/journal.pone.0189391)
Supplement: S1 File — http://dx.doi.org/10.1016/j.jneb.2016.04.343. (PDF) [file pone.0189391.s001.pdf]

NP17 Publication Patterns of PhD Students in the Illinois Transdisciplinary Obesity Prevention Program Versus Traditional Programs

Sharon Donovan, PhD, RD, sdonovan@illinois.edu, Department of Food Science & Human Nutrition, University of Illinois, 339 Bevier Hall, 905 South Goodwin Avenue, Urbana, IL 61801; *B. Fiese, PhD; J. Liechty, PhD; A. S. Keck, PhD, CCRP*

Objective: To compare publication patterns of doctoral students in a transdisciplinary research-based PhD/MPH degree program (I-TOPP) focused on child obesity prevention to students enrolled in a traditional PhD program within the same academic units.
Description: Publication records of I-TOPP (n=11) and traditional PhD (n=29) students were tracked from matched time of enrollment 2011-2013 to January 2016.
Evaluation: Number of peer-reviewed publications, year of publication, number of authors and departments per publication, and number of citations on Google Scholar were compared between I-TOPP and traditional students. Statistical differences were determined by t-test, $p<0.05$.
Conclusion and Implications: The I-TOPP students produced 4.5 ± 2.9 publications per student (49 total in 38 journals) compared to 2.1 ± 0.6 publications per student (61 total in 51 journals) produced by the traditional doctoral students, but this difference was not significant. Compared to traditional PhD students, I-TOPP students had more co-authors per publication (7.0 ± 0.4 vs. 5.3 ± 0.4, p =0.003), more departments represented per publication (3.4 ± 0.2 vs. 2.4 ± 0.2, p=0.002) and more organizations on each publication (2.35 ± 0.22 vs. 1.7 ± 0.1, $p<0.001$). To assess impact, the number of citations in Google Scholar was determined. The number of citations was 4-fold higher ($p<0.001$) per publication for I-TOPP than traditional PhD students (16.2 ± 3.7 vs. 3.1 ± 0.7). Findings suggest that transdisciplinary training does not negatively impact student publication records. Trandisciplinary trained scholars had publications with more diverse collaborations and higher impact than those from students in traditional doctoral programs.
Funding: USDA Grant #2011-67001-30101

NP18 Engaging Youth as Advocates to Create Healthy Snacking Zones Around Rural Schools – Year 4

Nancy Findholt, PhD, RN, findholt@ohsu.edu, Oregon Health & Science University, One University Boulevard, La Grande, OR 97850; *B. Izumi, PhD, MPH, RD,* Portland State University; *J. Shannon, PhD, RD, MPH; T. Nguyen, MD, PhD; C. Smith, MEd*

Objective: This on-going 4-year quasi-experimental study is evaluating the effects of an intervention that engages 4-H youth participants in advocacy for "healthy snacking zones" within 5 elementary/middle schools and nearby small food stores in a rural Oregon county.
Description: Youth have received nutrition education, participated in a photovoice assessment to build awareness of environmental barriers to healthy snacking and, with support from project staff and adult volunteers, have planned and implemented several outreach projects to promote healthy snacking within their schools and communities. Currently, club members are working with food store owners to increase availability and promotion of healthy snacks and beverages in stores near their schools.
Evaluation: Mixed methods are being used to evaluate intervention implementation, environmental change, and changes in children's snacking behaviors and BMI.
Conclusions and Implications: Preliminary results show a significant intervention effect on teachers' nutrition knowledge relevant to snacks (p=0.054), and decreased use of candy as rewards/incentives for students (p=0.005). Likewise, there have been improvements in students' snacking behavior, including statistically significant decreases in consumption of regular chips and sweetened fruit drinks (Cohen's d=0.06 and 0.03, respectively), and six food stores have designated shelving and refrigerator space that is stocked exclusively with foods and beverages that meet USDA Smart Snacks nutrition criteria. In conclusion, preliminary results suggest that youth advocacy is an effective strategy for promoting healthy snacking within rural schools and communities.
Funding: USDA Grant #2012-68001-19702

NP19 Optimizing a Childhood Obesity Prevention Program Using an Engineering-Inspired Framework

Lori Francis, PhD, lfrancis@psu.edu, Pennsylvania State University, 219 Biobehavioral Health Building, University Park, PA 16802; *R. BeLue, PhD; K. Kugler, PhD*

Objective: Effective programs to prevent obesity among preschool children have been largely unsuccessful. Using a novel approach to optimizing behavioral interventions, the multiphase optimization strategy (MOST), we will examine the effects of four intervention strategies over the course of 12 weeks on modifying the classroom environment in center-based childcare settings to increase vegetable intake, active play, and improve self-regulatory capacity in preschool children.
Description: MOST is an engineering-inspired framework that facilitates the selection of efficacious intervention components by first identifying and piloting the components, then assessing their individual and combined effects through efficient randomized experimentation. Following the principles of MOST we will identify one of the best combinations of intervention components in a thoughtful and systematic way. This is in contrast to the classical approach to intervention development with the 2-arm RCT, where even if the intervention is deemed efficacious, we would not know which components were efficacious and which were unnecessary. Thus using the principled framework of MOST, we increase the likelihood

Continued on page S119
